# DOT1L inhibitors block abnormal self-renewal induced by cohesin loss

**DOI:** 10.1038/s41598-021-86646-9

**Published:** 2021-03-31

**Authors:** Katelyn E. Heimbruch, Joseph B. Fisher, Cary T. Stelloh, Emily Phillips, Michael H. Reimer, Adam J. Wargolet, Alison E. Meyer, Kirthi Pulakanti, Aaron D. Viny, Jessica J. Loppnow, Ross L. Levine, John Anto Pulikkan, Nan Zhu, Sridhar Rao

**Affiliations:** 1 Blood Research Institute, Versiti, 8727 West Watertown Plank Road, Milwaukee, WI 53226 USA; 2 Department of Cell Biology, Neurobiology, and Anatomy, Medical College of Wisconsin, Milwaukee, WI USA; 3 Department of Natural Sciences, Concordia University Wisconsin, Mequon, WI USA; 4 Department of Medicine, Division of Hematology and Oncology, and Department of Genetics & Development, Columbia University Irving Medical Center, New York, NY USA; 5 Human Oncology and Pathogenesis Program, Leukemia Service, Department of Medicine, Department of Pathology, Molecular Cytology Core Facility, and Center for Epigenetics Research, Memorial Sloan Kettering Cancer Center, New York, NY USA; 6 Department of Pediatrics, Division of Hematology, Oncology, and Bone Marrow Transplantation, Medical College of Wisconsin, Milwaukee, WI USA

**Keywords:** Cancer, Cancer genetics, Epigenetics

## Abstract

Acute myeloid leukemia (AML) is a high-risk malignancy characterized by a diverse spectrum of somatic genetic alterations. The mechanisms by which these mutations contribute to leukemia development and how this informs the use of targeted therapies is critical to improving outcomes for patients. Importantly, how to target loss-of-function mutations has been a critical challenge in precision medicine. Heterozygous inactivating mutations in cohesin complex genes contribute to AML in adults by increasing the self-renewal capacity of hematopoietic stem and progenitor cells (HSPCs) by altering PRC2 targeting to induce *HOXA9* expression, a key self-renewal transcription factor. Here we sought to delineate the epigenetic mechanism underpinning the enhanced self-renewal conferred by cohesin-haploinsufficiency. First, given the substantial difference in the mutational spectrum between pediatric and adult AML patients, we first sought to identify if *HOXA9* was also elevated in children. Next, using primary HSPCs as a model we demonstrate that abnormal self-renewal due to cohesin loss is blocked by DOT1L inhibition. In cohesin-depleted cells, DOT1L inhibition is associated with H3K79me2 depletion and a concomitant increase in H3K27me3. Importantly, we find that there are cohesin-dependent gene expression changes that promote a leukemic profile, including *HoxA* overexpression, that are preferentially reversed by DOT1L inhibition. Our data further characterize how cohesin mutations contribute to AML development, identifying DOT1L as a potential therapeutic target for adult and pediatric AML patients harboring cohesin mutations.

## Introduction

Acute Myeloid Leukemia (AML) is a complex, high-risk myeloid malignancy which is caused by a diverse array of somatic mutations^1–3^. The overall prognosis for AML patients is poor, with 5-year survival lower than 50% in many populations. One common finding hindering the development of novel therapeutics is the large number (> 50) of mutations that contribute to AML. In recent years, several therapeutics have shown to be successful in a subset of patients that are positive for the specific mutation(s) they target^4^. However, not all mutations have a targeted therapy available, demonstrating the need to develop additional approaches. An alternative strategy is to target the downstream effects of disparate driver mutations that drive the leukemic program. *HOXA9* upregulation is commonly found in leukemia, occurring in patients with a variety of mutation(s) including *MLL*-rearranged, *NPM1*, *FLT3*, and *IDH* mutated leukemias^5,6^. Recently, genes encoding the cohesin complex (*RAD21*, *SMC1A*, *SMC3, STAG2*) have been identified as tumor suppressors in adult AML that ultimately result in *HOXA9* upregulation^3,7–14^. It is not known whether *HOXA9* upregulation can be identified in pediatric AML and given the significant genetic differences known to exist between adult and pediatric disease^15^, determining whether *HOXA9* upregulation is a common occurrence in pediatric disease could inform whether treatment strategies identified targeting *HOXA9* overexpression could be applicable for use in both pediatric and adult disease.

Heterozygous, loss of function mutations in the cohesin complex are thought to occur early during AML development and/or progression, implying that acquisition of a cohesin mutation is a critical event during leukemogenesis^12,16^. Although the canonical function of cohesin is to maintain sister chromatid cohesion during mitosis^17^, cohesin-mutated AML cells are rarely aneuploid beyond translocations. Further, our lab and others have shown that *Rad21* depletion does not lead to aneuploidy in vitro, collectively suggesting that defective chromosome segregation is unlikely to contribute to AML development^8–11^. In addition to its canonical role in mitosis, cohesin plays a critical role in gene expression by facilitating the interaction of distal *cis* regulatory elements with genes and the formation and/or maintenance of topology-associated domain (TAD) formation^18–21^. It is through these latter functions that cohesin mutations are thought to contribute to AML (reviewed in^3^). For example, our group reported that cohesin interacts with the Polycomb Repressive Complex 2 (PRC2) through CTCF and is necessary for proper silencing via trimethylation of H3K27 of the hematopoietic self-renewal transcription factors (TFs) *HoxA7* and *HoxA9*^11,22^. This is a distinct mechanism as compared to *MLLr* leukemias, where MLLr directly binds and activates expression of *HOXA* cluster genes^23–25^. Other studies demonstrated that haploinsufficiency of individual cohesin genes induces genome-wide changes in chromatin accessibility, resulting in an enrichment for binding sites of myeloid and hematopoietic stem and progenitor (HSPC) self-renewal TFs such as *ERG*, *RUNX1*, and *GATA2*, and *STAT5*^8–10^. Although these proposed mechanisms are distinct, collectively they conclude that disrupting cohesin function in HSPCs confers enhanced self-renewal by altering gene expression^21,26,27^. We hypothesized that the histone modifications that promote a leukemic transcriptional profile following cohesin loss may inform a targetable pathway in patients with cohesin mutations independent of other cooperating mutations. Further understanding of the mechanism of how loss of cohesin leads to these epigenetic changes is needed to target and reverse the effects of cohesin mutations. This is especially important in cohesin-mutated AML because cohesin mutations are likely present in the leukemic stem cell^9,10,16,28^.

Recently, a study showed that AF10, a member of the DOT1L histone modifying complex, can act as an epigenetic “reader”, capable of identifying genomic sites devoid of methylation on H3K27^29,30^. The absence of methylation on H3K27 at target genomic loci recruits the DOT1L complex via AF10 to methylate H3K79, resulting in H3K79me2 and the activation of gene expression^31^. In *MLLr*-leukemias this results in inappropriate expression of *HOXA9* but also other leukemias with high level *HOXA* cluster gene expression such as *NPM1*^*c*^^6^. Importantly, treating *MLLr* cells with DOT1L inhibitors reduces *HOXA9* expression, but does not induce a reaccumulation of the PRC2-mark H3K27me3 at the *HOXA9* promoter^25,32^. Instead, epigenetic silencing of *HOXA* cluster genes following DOT1L inhibition is mediated by SIRT1 via histone deacetylation^25,32^. Given the upregulation of *HOXA9* observed following loss of cohesin is through a distinct mechanism from *MLLr*^11^, we investigated the interplay between cohesin haploinsufficiency and DOT1L activity in promoting/driving a leukemic gene expression program. Using primary murine HSPCs as a model we demonstrate that a reduction in a core cohesin subunit (*RAD21* or SMC3) is associated with decreased H3K27me3 and increased H3K79me2, along with increased self-renewal capacity and a leukemic transcriptional profile. Inhibition of DOT1L in cohesin-depleted murine HSPCs restores normal self-renewal and gene expression, warranting future studies investigating the potential of DOT1L as a therapeutic target for cohesin-mutated AML.

## Materials and methods

### TARGET patient data

Data from NCI’s Therapeutically Applicable Research to Generate Effective Treatments (TARGET) study for AML^15^ were downloaded for 10 distinct patients with cohesin mutations (14 total datasets, 4 patients had data for both primary and recurrent disease) and 49 patients lacking cohesin mutations. We performed DESeq2 to identify differentially expressed genes and then Gene Set Enrichment Analysis on the differentially expressed gene profiles.

### Primary bone marrow isolation and viral transduction

All experimental protocols involving vertebrate animals were approved by the Medical College of Wisconsin Institutional Animal Care and Use Committee guidelines (IACUC# AUA00002688). All experiments were performed in compliance with the relevant guidelines for the care and use of mice as proscribed by the IACUC. Bone marrow was collected following euthanasia and HSPCs were isolated using a lineage depletion kit according to the manufacturer’s recommended protocol (Miltenyi, Cat# 130-090-858). HSPCs were cultured in StemPro medium (Gibco, Cat # 10640-019), IL-3 (10 ng/mL, Miltenyi 130-099-510), IL-6 (10 ng/mL, Miltenyi 130-096-684), and SCF (50 ng/mL, Miltenyi 130-101-698) for 16 h on suspension culture dishes. HSPCs were collected and transduced with lentivirus containing *Rad21* shRNA or Empty Vector (pLKO.1) control, similar to as previously described (Fig. 2A, details in^11^). Retronectin (Clontech, Cat# T100B) coated plates were preloaded with bone marrow medium (IDMD, 15% Fetal Bovine Serum, 1% Pen/Strep) containing *Rad21*-shRNA or empty vector constructs by centrifugation at 2000RPM for 60 min. Additional lentivirus was added to the HSPCs and they were transferred to the preloaded plates and centrifuged at 2000RPM for 90 min at 35 °C. The plates were incubated at 37 °C/5% CO2 for 16 h. Puromycin at 1 mcg/ml final concentration was added to select for transduced cells.

### Methylcellulose colony forming assays

Live, virally transduced HSPCs were plated in methocult medium (StemCell Technologies, Cat # 173434) containing puromycin (1 mcg/ml), and 10 µM DOT1L inhibitor (EPZ-4777 or EPZ-5676) or vehicle (DMSO) and incubated at 37 °C/5% CO2 for 7 days (as previously described^11^). Two independent researchers determined colony numbers and the average is reported. To start the next passage, the cells were collected in bone marrow wash buffer (HBSS, 1% Pen/Strep, 2% FBS), counted using a hemocytometer, and 1000 cells were plated into methocult containing puromycin (1 mcg/ml) and 10 µM DOT1L inhibitor (EPZ-4777 or EPZ-5676) or vehicle (DMSO). This process was repeated for all subsequent passages. Representative images included were taken at the end of passage 4.

### *Smc3* Mouse model bone marrow isolation

For sequencing studies, a mouse model of *Smc3* haploinsufficiency^10^ was used instead of lentiviral knockdown of *Rad21. Smc3*^+/+^ and *Smc3*^+/floxed^ mice were treated with polyI:polyC (pIpC; 7 doses, every other day) to induce excision beginning at 4 weeks of age. 4 weeks post completion of pIpC treatment, mice were euthanized and HSPCs were isolated as stated above. HSPCs were then plated at a density of 1,000 live cells (determined by Trypan blue staining) per plate in methocult with puromycin (1 mcg/ml) and 10 µM DOT1L inhibitor (Epizyme 5676 only) or vehicle (DMSO).

### Next generation sequencing approaches

Details for ChIP-sequencing (ChIPseq) and RNA-sequencing (RNAseq), including sample preparation, ERCC Spike-ins, and data analysis, can be found in the Supplemental material. Briefly, ChIPseq libraries were prepared at the end of primary passage. Cells were fixed followed by chromatin shearing and antibody-mediated isolation. Antibodies used can be found in Supplemental Table S2. Libraries were created using the NEBNext Ultra II DNA Library Prep Kit (NEB #E7645S). Sequencing was performed on an Illumina NextSeq (single-end, 75 cycles). RNA libraries were also prepared at the end of primary passage. After an ERCC-spike in, libraries were prepared using the NEB Ultra RNA library Prep Kit (#E7350). Sequencing was performed on an Illumina NextSeq (paired-end, 38 cycles each). Data can be found in GEO (Accession # GSE140361).

### Statistical analyses

Number of replicates and statistical methods used in each figure can be found in the corresponding figure legends. Indicators of significant *p* values are as follows: ^#^*p* < 0.1, *p < 0.05, **p < 0.01, ***p < 0.001.

## Results

### Children with cohesin-mutated AML have gene expression changes which may be reversible by DOT1L inhibitors

**Figure 1:**
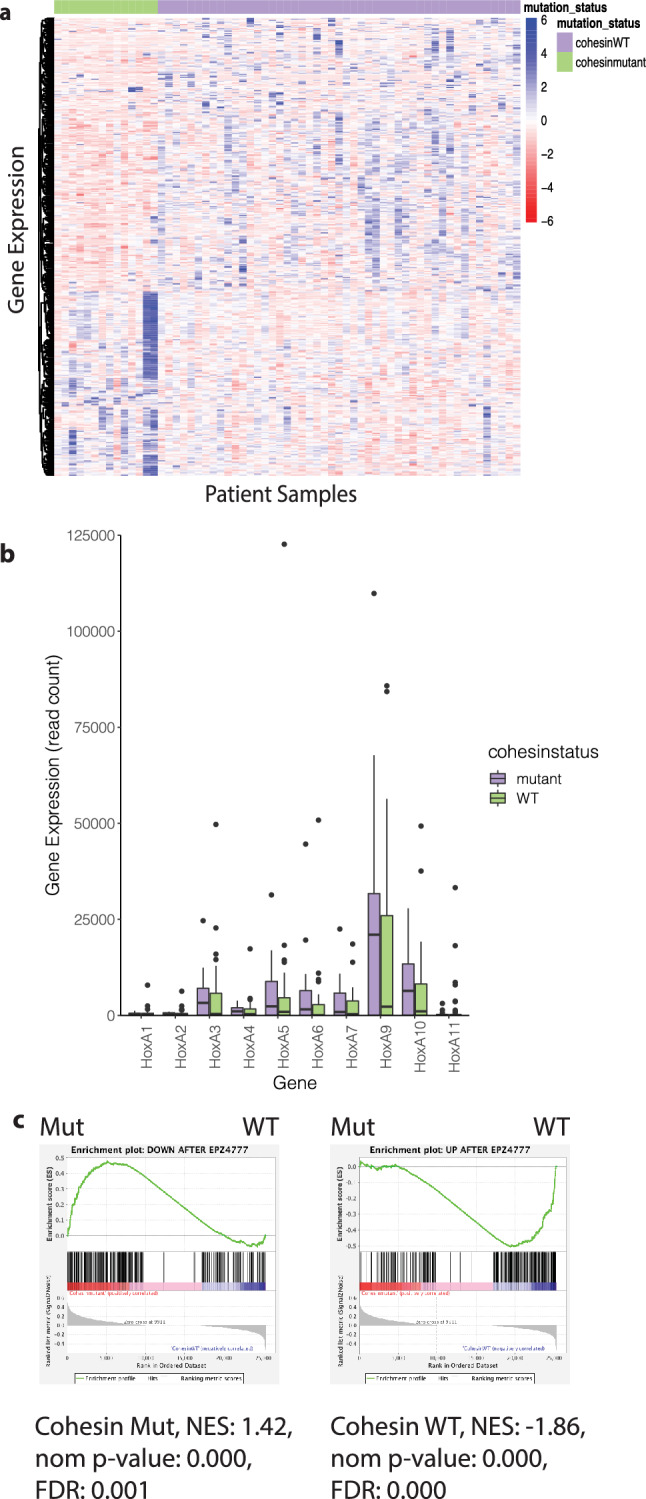
Pediatric AML patients with cohesin mutations have a transcriptomic signature which indicates they may be responsive to DOT1L inhibition. (**a**) Heatmap illustrating the clustering of cohesin-mutated patients (10 patients, 4 with primary and relapsed diseased, for a total of 14 samples) and cohesin-WT patients (49 patients). (**b**) Box and Whisker plots comparing TARGET AML expression data across the *HOXA* locus in cohesin mutant (n = 14) versus cohesin-WT (n = 49). According to the Wilcox test, significance is not reached for any gene. (**C**) GSEA analysis showing enrichment for a DOT1L inhibitor-derived signature.

Our prior work^11^ demonstrated that adult AML patients^12^ with cohesin mutations demonstrated high expression of *HOXA* cluster genes. As it has recently been shown by the NCI’s Therapeutically Applicable Research to Generate Effective Treatments (TARGET) study for AML, pediatric and adult AML are different diseases with distinct mutational spectra^15^. Adult AMLs typically have a higher mutational burden while pediatric AMLs contain more structural rearrangements^15^. In addition, cohesin mutations are enriched in *NPM1*^*c*^ mutations in adults, but in children *NPM1*-alerations are far less common. Given these differences, here we sought to determine if the common transcriptomic changes identified in adults (i.e. *HOXA* cluster upregulation) could also be identified in pediatric AMLs. We utilized publicly available RNAseq data from pediatric AML patients with (10 individual patients, 4 patients with both primary and recurrent disease data sets, n = 14) or without (n = 49) cohesin mutations and performed supervised clustering to investigate how cohesin-mutant patients cluster with each other compared to cohesin-WT patients. While the global differences in gene expression between the two groups were modest at best (Fig. 1a) we noted a trend towards elevated *HOXA9* expression in cohesin-mutant patients which did not reach statistical significance (Fig. 1b). Collectively, this indicates cohesin mutations in children do not overexpress *HOXA9* at high levels like adults, likely because of the differences in the spectrum of cooperating mutations between children and adult AML patients^2,12,15^.

While cohesin mutations don’t appear to be a major driver of *HOXA9* upregulation in pediatric leukemia, *HOXA9* upregulation still occurs fairly often in pediatric AML (such as in *MLLr* driven leukemias). Because adult AMLs which exhibit high level expression of *HOXA7/9* (such as *MLLr* and *NPM1*^c^) have been shown to respond to DOT1L inhibitors by suppressing *HOXA* cluster expression^25,30–35^, we wondered whether the *HOXA9* upregulation trend we observed might indicate that cohesin-mutated pediatric AML might be susceptible to DOT1L inhibition. We hypothesized that the gene expression changes induced by exposure of AML cells to DOT1L inhibitors would be inversely correlated with the transcriptome changes induced by cohesin-mutations. To address this question we reanalyzed published microarray data^36^ in which an AML cell line (MOLM-13) was exposed to the DOT1L inhibitor EPZ004777 in vitro to identify the 250 most up- or down-regulated genes as compared to vehicle, and used them as genesets in Gene Set Enrichment Analysis (GSEA)^37^. We observed a strong correlation between pediatric AMLs with cohesin mutations and the genes which were downregulated following DOT1L inhibitor treatment (Fig. 1c, left). In contrast, gene expression changes in cohesin wild-type AMLs correlate best with the genes which were upregulated following DOT1L inhibition (Fig. 1c, right). This data reveals that both DOT1L inhibition and cohesin haploinsufficiency affect an overlapping set of genes, indicating that use of DOT1L inhibitors in pediatric AML patients with *HOXA9*-upregulating mutations may be therapeutically advantageous. We therefore hypothesized the effects of cohesin haploinsufficiency on gene expression and self-renewal may be reversed by DOT1L inhibition.

### Abnormal self-renewal capacity following Rad21 depletion is rescued by DOT1L inhibition

**Figure 2:**
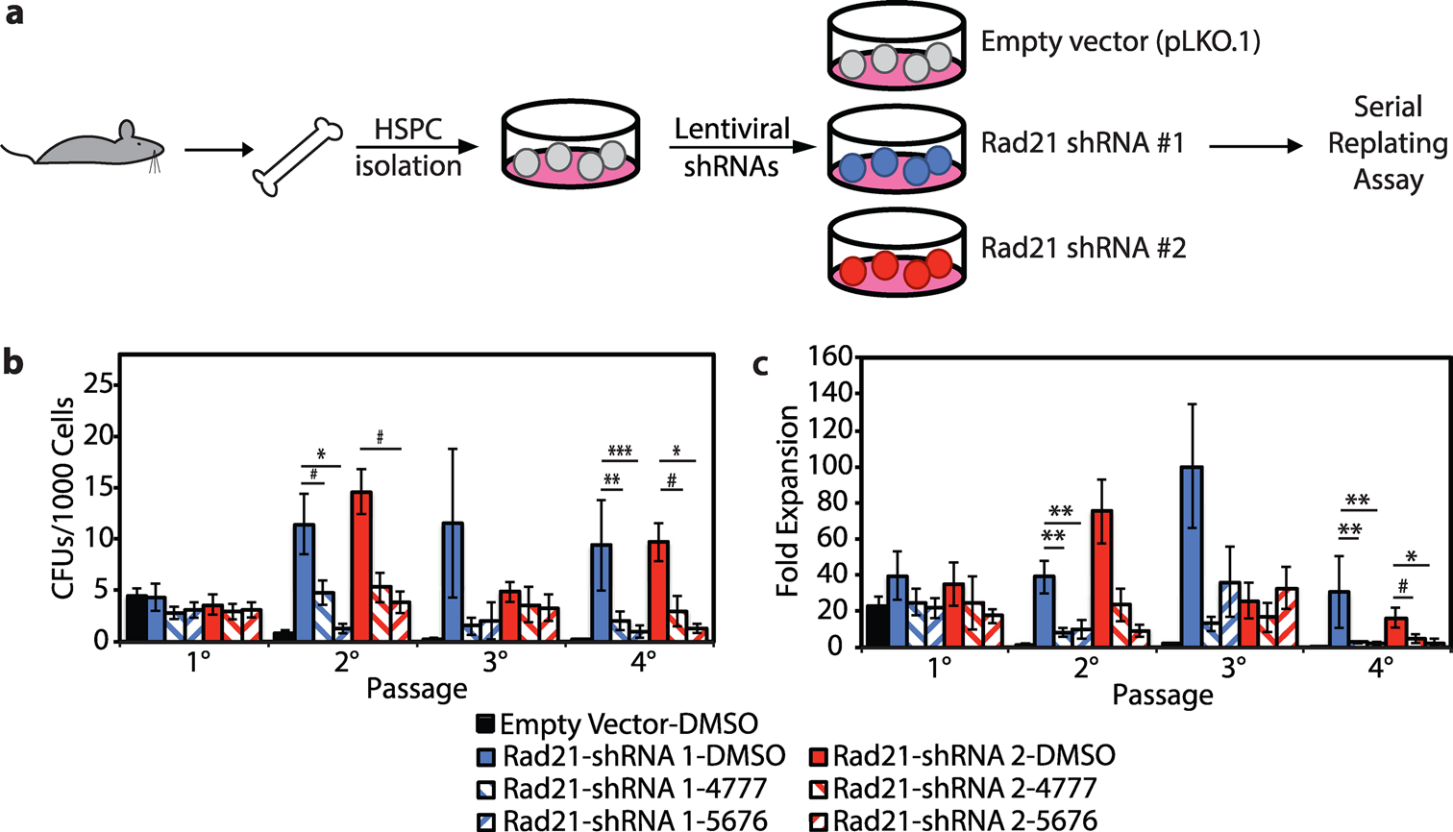
DOT1L inhibition rescues increased self-renewal phenotype of *Rad21* knockdown. (**a**) Scheme of experimental design. Hematopoietic stem and progenitor cells (HSPCs) are isolated from the bone marrow of mice and infected with lentiviral shRNAs (a pLKO empty vector (grey) or one of two *Rad21* shRNAs (red or blue), then the serial replating assay is performed. (**b**) Results of serial replating assay, displayed as number of colonies formed (CFUs) per 1000 cells plated for empty vector (black), *Rad21* shRNA #1 (blue) and *Rad21* shRNA #2 (red) treated with DMSO (solid) or DOT1L inhibitor EPZ4777 (forward slash) or EPZ5676 (backward slash). (**c**) Results of serial replating assay, displayed as fold expansion. B/C) n ≥ 2 per sample. Error bars represent the standard error of the mean. Statistical significance between individual data points determined by the Student’s T-test (two-tailed, unpaired). ^#^*p* < 0.1, *p < 0.05, **p < 0.01, ***p < 0.001.

To expand upon our hypothesis that DOT1L inhibition may be advantageous for cohesin-mutated AML, we wanted to test directly if DOT1L inhibitors could block the abnormal self-renewal induced by cohesin mutations, which is dependent on elevated expression of *HOXA9*^11^. To test this, we first performed in vitro serial replating assays (Fig. 2a) to assess the effects of DOT1L inhibition on self-renewal capacity in murine HSPCs depleted for the cohesin subunit *Rad21*. This model has been previously shown by our lab to reduce both mRNA and protein levels of RAD21 to 50%^11^. The DOT1L inhibitors used, EPZ-4777 and EPZ-5676, were developed by Epizyme. The latter (EPZ-5676), also known as pinometostat, has shown modest effects in early clinical trials to reduce H3K79 methylation and prolong survival in adult acute leukemia patients^38^.

As we have shown previously^11^, cohesin loss leads to increased self-renewal in serial replating assays in vitro both in terms of colony-forming units (CFUs; Fig. 2b) and proliferation (Fold Expansion; Fig. 2c). Newly, our results demonstrate that treatment of *Rad21*-depleted cells with DOT1L inhibitors (slashed bars) significantly reduces the enhanced self-renewal capacity of cohesin-depleted cells (Fig. 2b,c), while having minimal effect on empty vector controls (Supplemental Fig. S1). We observed that EPZ-5676 (back-slashed bar) has a more robust effect than EPZ-4777 (forward-slashed bars) in terms of suppressing abnormal self-renewal. While neither inhibitor completely abolishes the enhanced self-renewal induced by cohesin depletion, statistically significant differences are seen at passage 4 (Fig. 2b,c). Images at the end of quaternary passage show the strong effect of DOT1L inhibition on colony formation, as the DMSO-treated condition shows formation of mostly CFU-GM colonies, which are absent under either inhibitor (Supplemental Fig. S1). We observe the same reversal of self-renewal capacity by DOT1L inhibition using a genetic model of *Smc3* haploinsufficiency (*Smc3*het^10^), another core cohesin complex member (Supplemental Fig. S2). Taken together these data indicate that the abnormal self-renewal phenotype conferred by either RNAi-mediated depletion of *Rad21* or genetic haploinsufficiency for *Smc3* is reversed by exposure to two different DOT1L inhibitors, with pinometostat (EPZ-5676) being quantitatively more effective, especially in the *Smc3* model.

### DOT1L inhibition leads to H3K79me2 depletion and reduction of *HoxA* expression in cohesin-deficient cells

**Figure 3:**
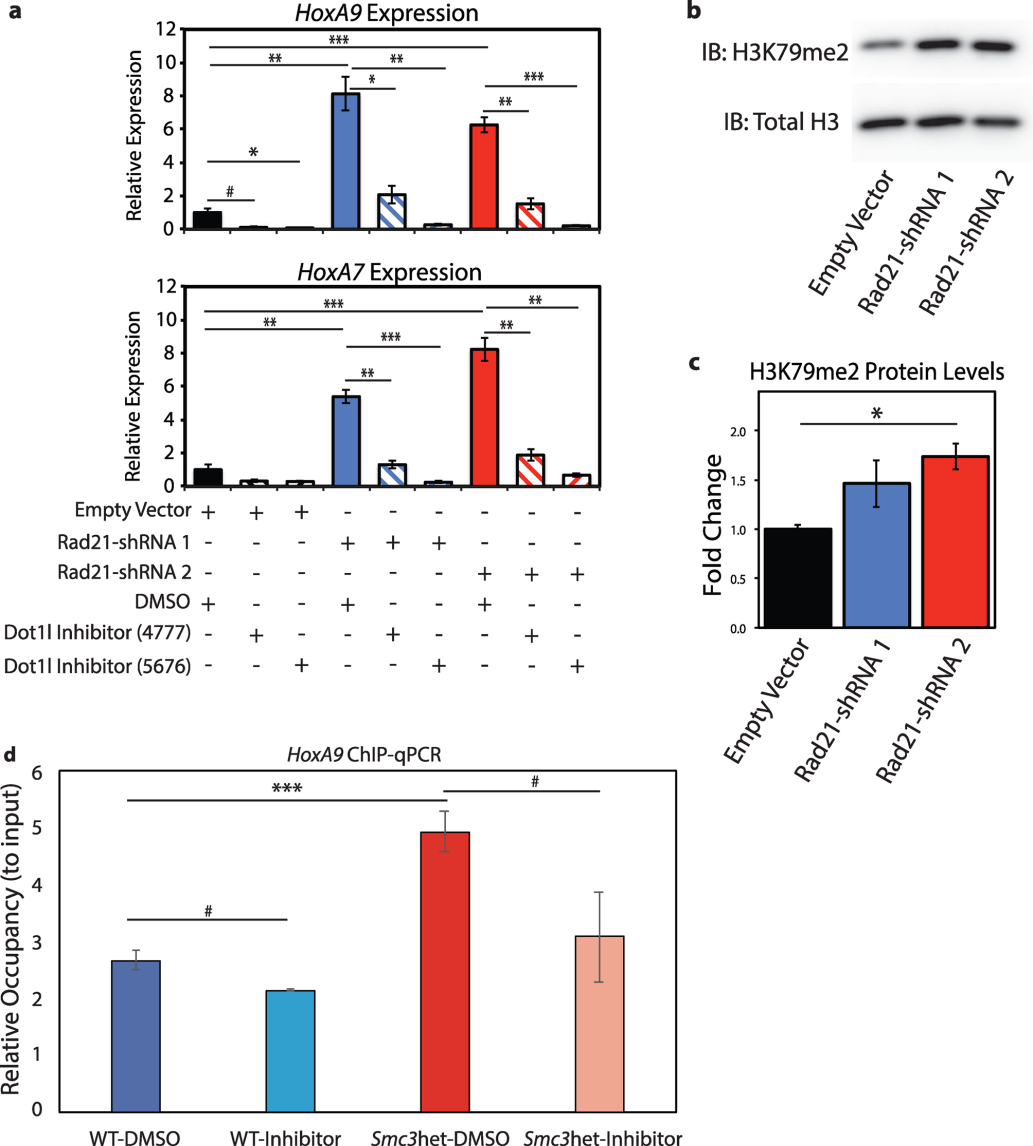
*Rad21* depletion leads to a global increase in H3K79me2 that is correlated with increased *HoxA9* and *HoxA7* expression and decreases upon DOT1L inhibition. (**a**) Expression of *HoxA9* and *HoxA7* determined via RT-qPCR, in empty vector and *Rad21* shRNA infected cells both untreated and treated with 2 DOT1L inhibitors (EPZ4777 and EPZ5676). n = 3. Error bars represent the standard error of the mean and statistical significance between data points was determined using Student’s T-test (two tailed, unpaired). *p < 0.05, ^ł^*p* < 0.01. (**b**) Western blot probing for H3K79me2 and total H3 in empty vector and *Rad21* shRNA infected cells. (**c**) Quantification of western blot, n = 2 for empty vector and shRNA2, n = 3 for shRNA1, ^#^*p* < 0.1, *p < 0.05, **p < 0.01, ***p < 0.001 (**d**) Relative H3K79me2 occupancy at the *HoxA9* locus identified via ChIP-qPCR. n = 2–5, ^#^*p* < 0.1, *p < 0.05, **p < 0.01, ***p < 0.001.

After identifying the reduction in self-renewal that results from DOT1L inhibition on cohesin-depleted cells, we sought to determine the mechanism by which DOT1L inhibition reverses the phenotype of cohesin knockdown. Our previous work identified that *Rad21*-depletion leads to an increase in *HoxA7/9* expression conferring abnormal self-renewal^11^. We therefore hypothesized that DOT1L inhibition would reduce *HoxA7/9* expression in cohesin-depleted cells as it does in the setting of other driver oncogenes^31,33^. To test this hypothesis, we measured *HoxA7* and *HoxA9* levels via RT-qPCR, primer sequences can be found in Supplemental Table S1. We observed that in the presence of DOT1L inhibitors, *HoxA7*/9 increases following *Rad21* knockdown were significantly blunted (Fig. 3a). Given the critical role of *HoxA7/9* in abnormal self-renewal following cohesin-loss^11^, the reduced expression of these genes following DOT1L inhibition accounts for the reduced self-renewal phenotype (Fig. 2).

Based on recent works demonstrating that AF10 acts as an epigenetic reader for unmethylated H3K27^25,29,32,34^, we hypothesized that the global decrease in H3K27me3 we observed following cohesin-loss^11^ may be accompanied by a corresponding global increase in levels of the DOT1L-dependent mark H3K79me2. To test this hypothesis we measured global H3K79me2 in *Rad21*-depleted cells compared to the empty vector by western blot and observed a global increase in total H3K79me2 levels in *Rad21*-depleted cells (Fig. 3b,c). We also identified that treatment with DOT1L inhibitors rescues the increased H3K79me2 that results from *Rad21*-depletion via western blot (Supplemental Fig. S3). To take these observations a step further, we performed ChIP-qPCR (primers sequences can be found in Supplemental Table S1) for H3K79me2 at *HoxA9* in cells that are heterozygous for *Smc3*^10^ (Supplemental Fig. S2), a genetic model that more accurately recapitulates what is found in human disease and avoid the pitfalls of shRNAS such as multiplicity of infection. Adding to our global H3K79me2 analysis, we show that at the *HoxA9* locus, H3K79me2 is increased upon cohesin haploinsufficiency and subsequently reduced by DOT1L inhibition (Fig. 3d). In fact, the increased H3K79me2 resulting from cohesin haploinsufficiency returns to levels comparable to both WT-DMSO and WT-Inhibitor conditions upon DOT1L inhibition (*p* values > 0.2). No significant H3K79me2 differences are observed at the *GAPDH* promoter (Supplemental Figure S3). Collectively, this indicates that DOT1L inhibitors can reverse the increased H3K79me2 observed following cohesin loss, both globally and at the *HoxA9* locus. Interestingly, depletion of H3K79me2 occurs in both WT and cohesin-deficient conditions following DOT1L inhibition, indicating the effect of the DOT1L inhibitors is substantial but appears to have a greater effect in cohesin-deficient cells. However, how these epigenetic changes affect gene expression in WT and cohesin-mutant cells and whether the transcriptomes of WT and cohesin-mutant cells respond in the same manner to DOT1L inhibition is unknown.

### DOT1L inhibition demonstrates activation of differentiation-associated gene expression programs in cohesin-deficient cells

We hypothesized that DOT1L inhibition would broadly affect the transcriptome in cells and provide mechanistic insights into how loss of DOT1L activity affects cohesin haploinsufficient HSPC differentiation and proliferation. To test this, we used a genetic model of *Smc3* haploinsufficiency (Supplemental Fig. S2) and the more potent DOT1L inhibitor (EPZ-5676). We performed RNA-sequencing (RNAseq) followed by differential expression analysis to identify global gene expression changes in *Smc3*het versus WT-HSPCs^39^. Consistent with our RT-qPCR data, we observed a profound increase in *HoxA9* expression in *Smc3*het compared to WT HSPCs that was substantially reduced by DOT1L inhibition (Supplemental Fig. S4). Importantly, the reduction of *HoxA9* expression upon DOT1L inhibition is stronger in the cohesin-haploinsufficient background than the wild-type, a pattern which correlates with the H3K79me2 occupancy identified at the *HoxA9* locus (Fig. 3d). Collectively, this indicates that DOT1L inhibition reduces *HoxA9* expression in cohesin-haploinsufficient cells via reduction of H3K79me2 at the *HoxA9* locus.

**Figure 4:**
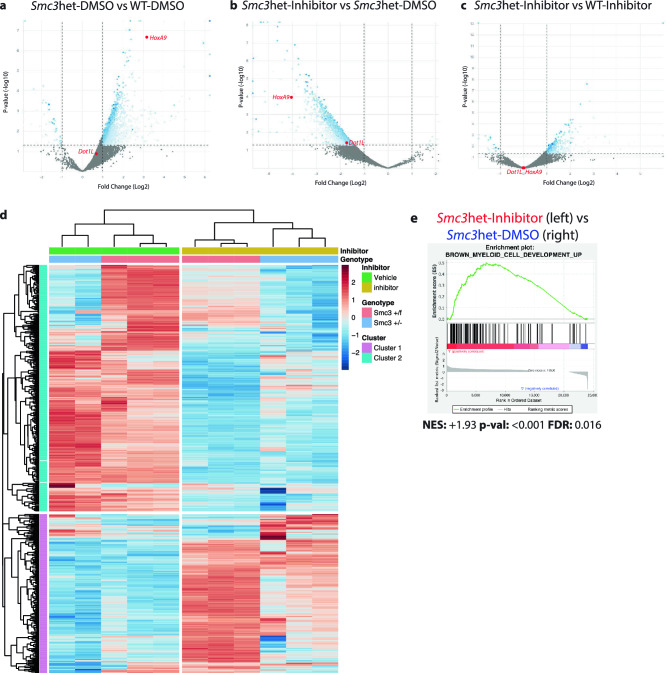
RNAseq identifies reversion of the global transcriptome and a differentiation signature following DOT1L inhibition. (**a**–**c**) Volcano plots representing gene expression in the following comparisons: *Smc3*het-DMSO vs WT-DMSO (**a**), *Smc3*het-Inhibitor vs *Smc3*het-DMSO (**b**), and *Smc3*het-Inhibitor vs WT-Inhibitor (**c**). Red dots indicate *HoxA9* and *DOT1L* expression for reference. (**d**) Heatmap illustrating the expression of differentially regulated genes across all 4 conditions. (**e**) GSEA plot of a geneset for Myeloid Development, with corresponding statistics below.

Similar to previous reports^9,10,28^, cohesin haploinsufficiency leads to a robust transcriptome-wide increase in gene expression (2667 upregulated and 57 downregulated genes), consistent with widespread gene de-repression (Fig. 4a). In the *Smc3*het background, DOT1L inhibition causes predominantly gene repression (194 upregulated and 401 downregulated genes, Fig. 4b), consistent with DOT1L being critical to inducing and/or maintaining gene expression in HSPCs haploinsufficient for cohesin. Importantly, when comparing the transcriptomic differences between the *Smc3*het and WT conditions (Fig. 4a vs. Fig. 4c), DOT1L inhibition dramatically reduces the number of differentially expressed genes (302 upregulated and 28 downregulated genes, Fig. 4c), with a more prominent effect on the genes that are upregulated (2667 to 302 upregulated genes, a 89% reduction) than downregulated genes (57 to 28 downregulated genes, a 51% reduction). This confirms that there are a subset of cohesin-dependent gene expression changes which are more sensitive to DOT1L inhibition (likely genes that are upregulated by cohesin haploinsufficiency but downregulated upon DOT1L inhibition). Thus, consistent with our self-renewal data (Fig. 2, Supplemental Figs. S1 and S2), cohesin-haploinsufficient cells are more sensitive to gene expression changes following DOT1L inhibition than wild-type cells.

We next hypothesized that DOT1L inhibition would globally revert the transcriptome of cohesin haploinsufficient cells to be me more similar to WT cells. To test this we performed unsupervised clustering using the top 500 most variable genes across all samples (Fig. 4d). Clustering analysis reveals that the DOT1L inhibitor has a stronger effect on gene expression than the loss of one *Smc3* allele, since the conditions first cluster by treatment rather than genotype (Fig. 4d). Further, we identified two distinct clusters of genes which demonstrated inverse patterns of expression, with Cluster 1 (Purple, ≈lower third, Fig. 4d) genes being upregulated in inhibitor-treated cells and the Cluster 2 (Aqua, ≈upper 2/3, Fig. 4d) representing genes that are downregulated after DOT1L inhibition. To identify global pathways which may differ between the two clusters we identified enriched pathways using PANTHER^40^. Among the top ten GO terms based upon fold enrichment was an enrichment for differentiation-associated pathways in the upregulated Cluster 1 (≈sevenfold, Supplemental Fig. S4), suggesting that DOT1L inhibitor treatment may promote differentiation of cells away from an HSPC-like state.

To further delineate the pathways perturbed by DOT1L inhibition in *Smc3*het cells, we utilized GSEA with curated genesets (MSigDB v7.0, C2 collection) and identified a total of 19 which exhibited statistically significant enrichment (*p* val < 0.01, FDR < 0.05) in the *Smc3*het cells following DOT1L inhibitor treatment (Supplemental Table S3). Among the genesets enriched in the DOT1L-inhibited cells were three different signatures associated with myeloid cell differentiation (Fig. 4e, Supplemental Fig. S4). This is also consistent with our self-renewal data, as DOT1L inhibition slowed growth of cohesin-mutant cells which is also suggestive of differentiation. Collectively, this work further substantiates that DOT1L inhibition in cohesin haploinsufficient cells induces differentiation by blocking *HoxA9*-dependent target genes.

### DOT1L inhibition restores the PRC2-mark H3K27me3 at a subset of genomic loci

**Figure 5:**
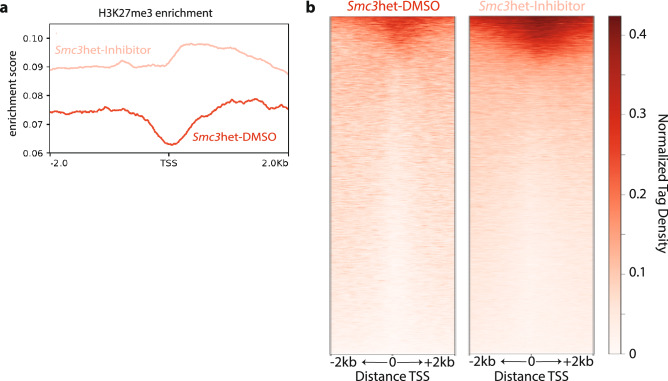
Genome wide changes in H3K27me3 parallel changes identified at the *HoxA9* locus. (**a**) Enrichment plots depicting H3K27me3 coverage +/− 2 kb of the TSS (x-Axis), with the Y-axis indicating enrichment score. (**b**) Heatmap visualizing genome wide H3K27me3 in the *Smc3*het-DMSO (red, left) and *Smc3*het-Inhibitor (pink, red). The X-axis represents all genes identified in this population across the mm10 genome. Each gene corresponds to one row, with 2 kb up and downstream of the TSS shown. The Y-axis represents normalized Tag density.

Our prior work revealed increased global H3K27me3 upon cohesin loss^11^, and here we demonstrate that cohesin loss also leads to an increase in global H3K79me2 that can be reduced by DOT1L inhibition. While it has been published that DOT1L inhibition does not induce an accumulation of H3K27me3 at the *HOXA* cluster^32^, given the global changes in H3K27me3 and H3K79me2 we have observed here (Fig. 3, Supplemental Fig. S3) and in prior studies^11^, we wondered where within the genome H3K27me3 accumulated following treatment with EPZ-5676. To address this we performed chromatin immunoprecipitation coupled with next generation sequencing (ChIPseq) for the PRC2-mark H3K27me3 in *Smc3*het cells with or without DOT1L inhibitor (EPZ5676) treatment. We did not perform ChIPseq for H3K79me2 because of the near complete loss of H3K79me2 with DOT1L inhibition (Supplemental Fig. S3) leading to unreliable ChIPseq signals. We were surprised to find that H3K27me3 did not reaccumulate at the TSSs of *HoxA7/9* (data not shown)*.* To identify genome-wide changes in H3K27me3, we queried our ChIPseq for H3K27me3 within 2 kb of the TSS of all well-annotated gene promoters and observed DOT1L inhibition rescues H3K27me3 in cohesin haploinsufficient cells genome-wide (Fig. 5a), at a substantial fraction of genes (Fig. 5b, upper third). Importantly, the majority of genes that lack H3K27me3 in *Smc3*het cells showed virtually no change following DOT1L inhibition (Fig. 5b, lower two thirds), indicating that DOT1L inhibition did not induce aberrant H3K27me3 deposition at these genomic loci. As such, it supports a model whereby the dynamic relationship between the DOT1L mark H3K79me2 and the PRC2 mark H3K27me3 are at a minority of loci.

While the PRC2-mark H3K27me2 is associated with gene silencing, alterations in the PRC2-mark may be compensated by other changes (i.e. DNA methylation and histone acetylation) which prevent substantial changes in gene expression^41–43^. To address this issue, we compared the Log2Fold Change (FC) for both the mRNA levels and H3K27me3 ChIP-seq signal within a 250 bp window around the TSS of all RefSeq promoters (Supplemental Fig. S5). Not surprisingly, the majority of changes were small, with minimal correlation between the changes in H3K27me3 and mRNA changes. Importantly, even when only genes with significant RNAseq changes (*p* val < 0.05, |Log2FC|> 1) were visualized (red dots in Supplemental Figure S5) it became apparent that more genes with a significant increase in H3K27me3 showed an increase in mRNA levels (65) versus those that demonstrated reduced expression (48). Collectively, these results indicate there is little direct correlation between the deposition of H3K27me3 and gene expression, as has been seen in other situations^44–46^. While DOT1L inhibition does revert the transcriptome of cohesin heterozygous cells to a more WT state (Fig. 4), the subsequent changes in the PRC2-mark H3K27me3 are unlikely to be a critical driver. In fact, H3K79me2 levels are more likely informative regarding gene expression changes, and suggest that other compensatory epigenetic changes are occurring in the cohesin-mutant context^32^ and will need to be identified to fully explain the changes in gene expression we have identified.

## Discussion

AML is a genetically heterogeneous disease, with recent progress being made in the area of targeted therapeutics^47,48^. However, most successful targeted therapeutics are directed at activating oncogenic mutations such as *FLT3-ITD*. Much less progress has been made for targeting loss-of-function (i.e. tumor suppressor) mutations such as those that disrupt the cohesin complex^49^. Here we investigate the possibility of targeting DOT1L as a potential therapeutic strategy for patients with cohesin mutations and propose that targeting the downstream epigenetic effects of cohesin loss may be beneficial for patients with cohesin mutations.

**Figure 6:**
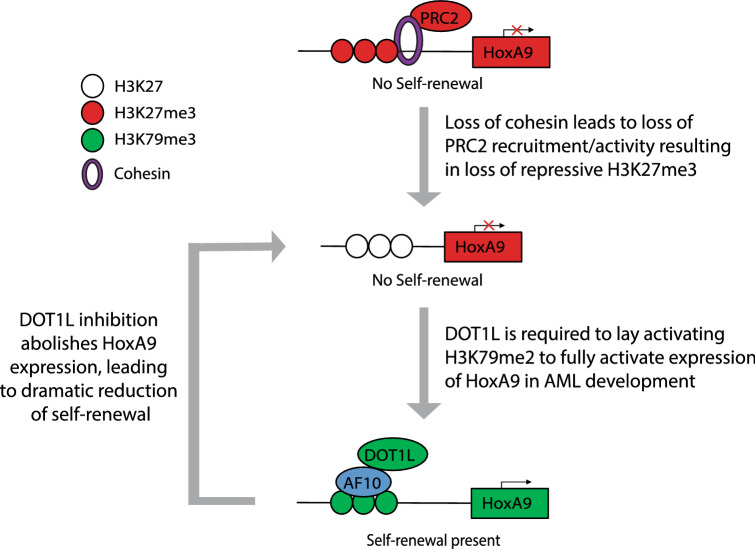
Mechanistic model for how *HoxA9* expression is regulated during leukemogenesis and DOT1L inhibition.

Our previous work has demonstrated that adult patients with cohesin mutations overexpress the self-renewal genes *HOXA7/9*^11^. To extend this observation to pediatric AML, we uncovered a transcriptional signature indicating that cohesin-mutated patients from the TARGET database^15^ may be sensitive to DOT1L inhibition. Subsequently, we demonstrated that both the increased self-renewal and elevated *HoxA9* expression phenotypes caused by loss of cohesin are reversible by DOT1L inhibition. Additionally, we have identified that cohesin loss leads to an increase in H3K79me2 that can be rescued to WT levels with DOT1L inhibition. Advancing our previous work that there is a global decrease in H3K27me3 upon cohesin loss, we identified that the loss of the PRC2-mark can be also rescued at a subset of genomic loci following DOT1L inhibition, indicating there is a dynamic interplay between the DOT1L and PRC2 complexes at certain loci. Our work here leads us to propose a mechanism for how leukemic transcriptional profiles (i.e. *HOXA9* expression) can be targeted (Fig. 6). We propose that upon loss of cohesin, likely through decreased PRC2 activity and/or recruitment, the H3K27me3 landscape is reduced, leaving histones without repressive marks accessible to the activity of DOT1L. Only after DOT1L lays down H3K79me2 is *HOXA9* expression fully activated. Our studies here outline how the inhibition of DOT1L leads to a reversal of the transcriptomic changes induced by loss of cohesin and subsequent *HOXA9* activation, resulting in a transcriptomic signature consistent with increased differentiation (Fig. 4, Supplemental Fig. S4).

We are not the first group to investigate the efficacy of DOT1L inhibition in AML. Kuhn et al.recently showed the effectiveness of DOT1L inhibition in combination with inhibition of menin-MLL in *NPM1*^*c-*^ driven leukemia^31^. Additionally, DOT1L inhibition in the context of *MLLr* leukemia has also been identified as a potential therapeutic strategy^25,30,32–36,38,50–53^. Here, our studies uniquely look at the effects of DOT1L inhibition in the context of isolated cohesin haploinsufficiency, allowing us to identify the specific mechanism behind the interplay between cohesin loss and DOT1L inhibition. However, given that all three driver mutations (MLL-AF9, *NPM1*^*cA*^, and cohesin haploinsufficiency) induce *HOXA9* expression, one hypothesis is that DOT1L inhibitors would be effective against any AML with high-level expression of *HOXA9*^25^*,* including the cohesin-mutated pediatric AMLs we examined above. This is appealing because approximately half of AML specimens overexpress *HOXA9*^6^. Therefore, the number of genetic mutations that could be potentially be targeted by pinometostat may be more expansive even though these mutations operate through distinct mechanisms. Being able to target downstream pathways that become altered due to a variety of genetic lesions is an exciting concept, as therapeutics already in use could possibly be expanded to include additional patient populations, which would allow us to more rapidly improve the poor survival of AML patients. Importantly, our work focused on murine cells as a model because of their well-defined genetic nature. Given this, critical studies need to be performed in primary, cohesin-mutated AML samples to identify if DOT1l inhibition is a potential therapy for this group of patients.

One important distinction between *MLLr*-leukemias and cohesin-loss is the mechanism by which they induce *HOXA* overexpression. MLLr proteins can directly bind to the *HOXA* cluster to regulate gene expression^25^, whereas loss of cohesin appears to disrupt proper targeting of the PRC2 complex^3,11,43^. Thus, the fact that either mutation can be targeted by pinometostat points to the critical need for DOT1L in the activation of *HOXA* genes. In addition, given the role cohesin plays in nuclear architecture, it is not surprising that inhibition of DOT1L did not completely revert the genomic localization of H3K27me3, and its reaccumulation did not correlate with gene expression. One can speculate that loss of cohesin causes a fundamental change in chromatin architecture, preventing proper PRC2 targeting even with DOT1L inhibition. As such, it is likely that alternative epigenetic pathways, such as DNA methylation, histone acetylation, or perhaps even nucleosome remodeling are what ultimately induce the gene expression changes observed following DOT1L inhibition. Regardless of the exact mechanism, we observed both a reversion in the transcriptome and an increase in the differentiation signature in cohesin-deficient cells upon DOT1L inhibition, underscoring the therapeutic potential of these small molecules.

In sum, our work shows that the efficacy of DOT1L inhibition should continue to be investigated in the context of patients with cohesin mutations and *HOXA9* upregulation. Studies investigating the effects of pinometostat in primary AML samples, with cohesin mutations and/or HOXA9 upregulation, will be beneficial in determining the true efficacy of DOT1L inhibition.

## Supplementary Information

Supplementary Information
