# Supplementary material for: DOT1L inhibitors block abnormal self-renewal induced by cohesin loss
Source: Sci Rep. 2021 Mar 31;11:7288. doi: 10.1038/s41598-021-86646-9 (PMC8012605; doi:10.1038/s41598-021-86646-9)
Supplement: Supplementary file 1 — Supplementary Information [file 41598_2021_86646_MOESM1_ESM.pdf]

DOT1L inhibitors block abnormal self-renewal induced by cohesin loss

Katelyn E. Heimbruch[1,2,+], Joseph B. Fisher[1,3,+], Cary T. Stelloh[1], Emily Phillips[1], Michael H. Reimer Jr. [1,2], Adam J. Wargolet[3], Alison E. Meyer[1], Kirthi Pulakanti[1], Aaron D. Viny[4], Jessica J. Loppnow[3], Ross L. Levine[5], John Anto Pulikkan[1], Nan Zhu[1,2], and Sridhar Rao[1,2,6,*]

[1]Blood Research Institute, Versiti, Milwaukee, WI
[2]Department of Cell Biology, Neurobiology, and Anatomy, Medical College of Wisconsin, Milwaukee, WI
[3]Department of Natural Sciences, Concordia University Wisconsin, Mequon, WI, USA
[4]Department of Medicine, Division of Hematology and Oncology, and Department of Genetics & Development, Columbia University Irving Medical Center, New York, NY, USA
[5]Human Oncology and Pathogenesis Program, Leukemia Service, Department of Medicine, Department of Pathology, Molecular Cytology Core Facility, and Center for Epigenetics Research, Memorial Sloan Kettering Cancer Center, New York, NY
[6]Department of Pediatrics, Division of Hematology, Oncology, and Bone Marrow Transplantation, Medical College of Wisconsin, Milwaukee, WI

[+] These authors contributed equally to this work.

* corresponding author:
Sridhar Rao
Blood Research Institute
Versiti
8727 West Watertown Plank Road
Milwaukee, WI 53226
414-937-3841
Fax 414-937-6284
sridhar.rao@versiti.org

Supplemental Figure 1

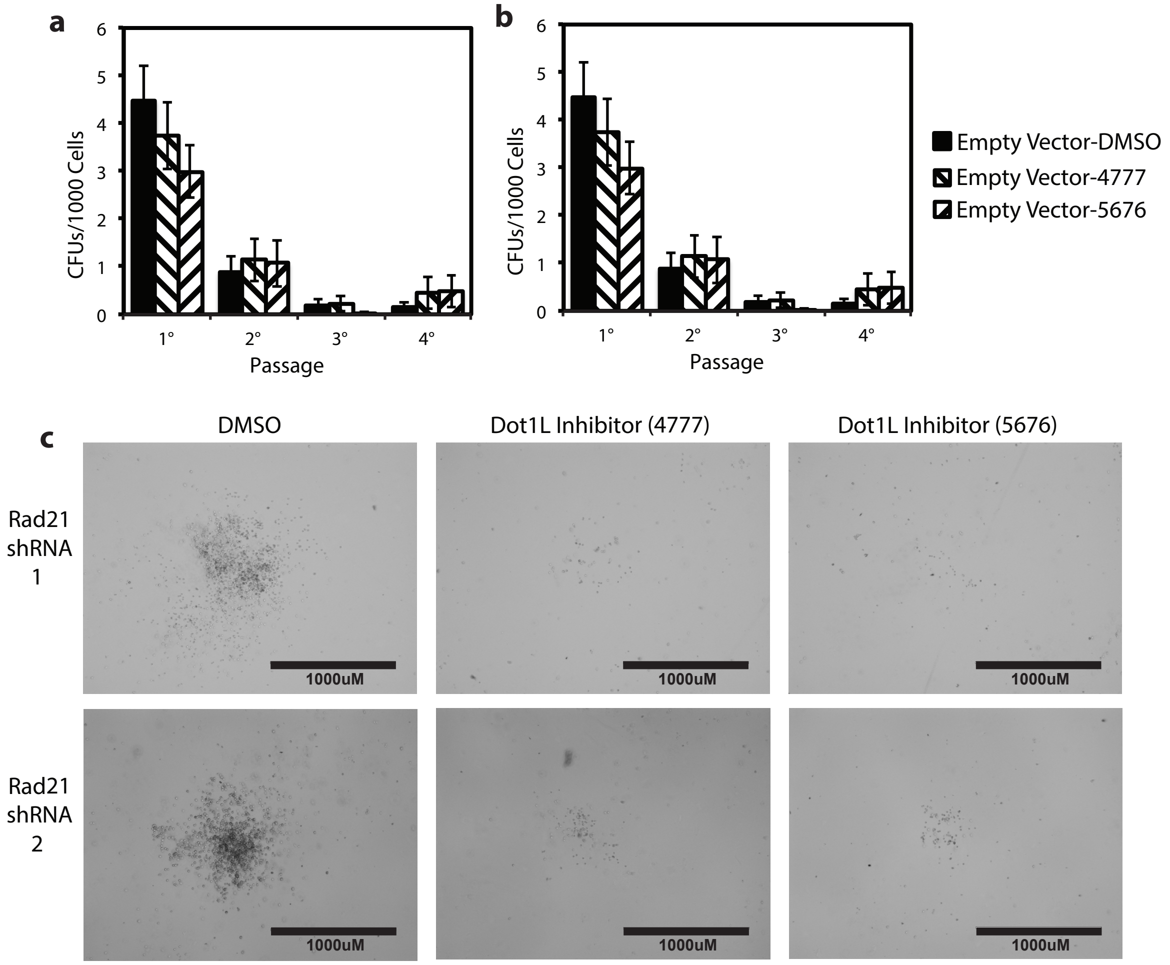

Supplemental Figure 1: **a)** Results of serial replating assay for empty vector controls treated with DOT1L inhibitor displayed as colony forming units (CFUs). No statistically significant differences determined by Student's T-test (unpaired, two-tailed) were observed. **b)** Results of serial replating assay for empty vector controls treated with DOT1L inhibitor displayed as fold expansion. a&b) n = 4 for each data point. Error bars represent the standard error of the mean, and statistical significance between individual data points was determined using Student's T-test (two tailed, unpaired). No significance differences was observed in any pairwise combination. **c)** Images of colonies formed during the serial replating assay taken at the end of quaternary passage.

Supplemental Figure 2

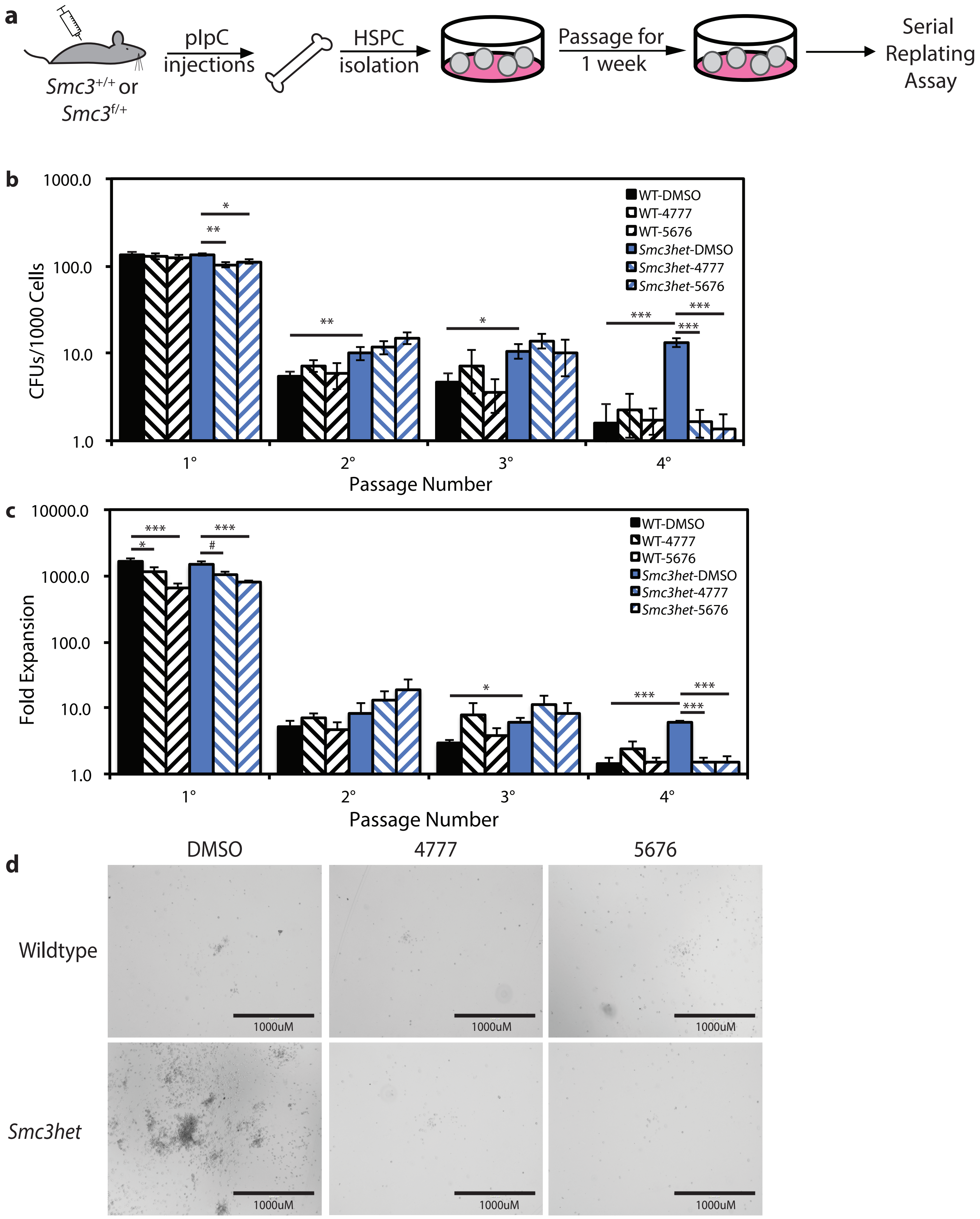

Supplemental Figure 2. a) Experimental procedure for cohesin haploinsufficient mouse model in vitro experiments. **b-d)** Results of serial replating assay with cells heterozygous for Smc3, displayed as colony forming units (b), fold expansion (c), and images of individual colonies formed at the end of quaternary passage. (d). n = >2 for each data point. Error bars represent the standard error of the mean, and statistical significance between individual data points was determined using Student's T-test (two tailed, unpaired). # = $p < 0.1$, * = $p < 0.05$, ** = $p < 0.01$, *** = $p < 0.001$

Supplemental Figure 3

a

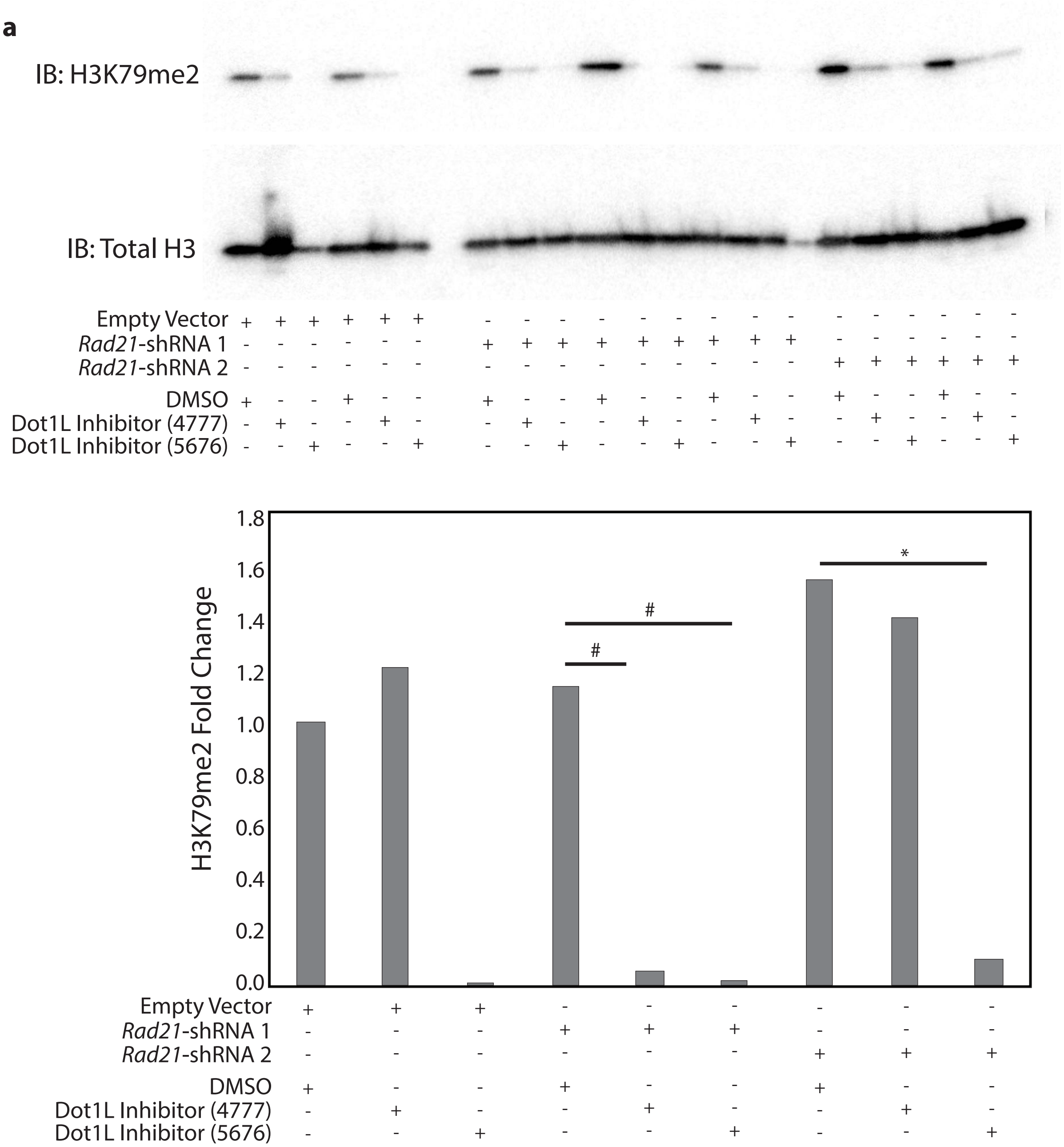

b

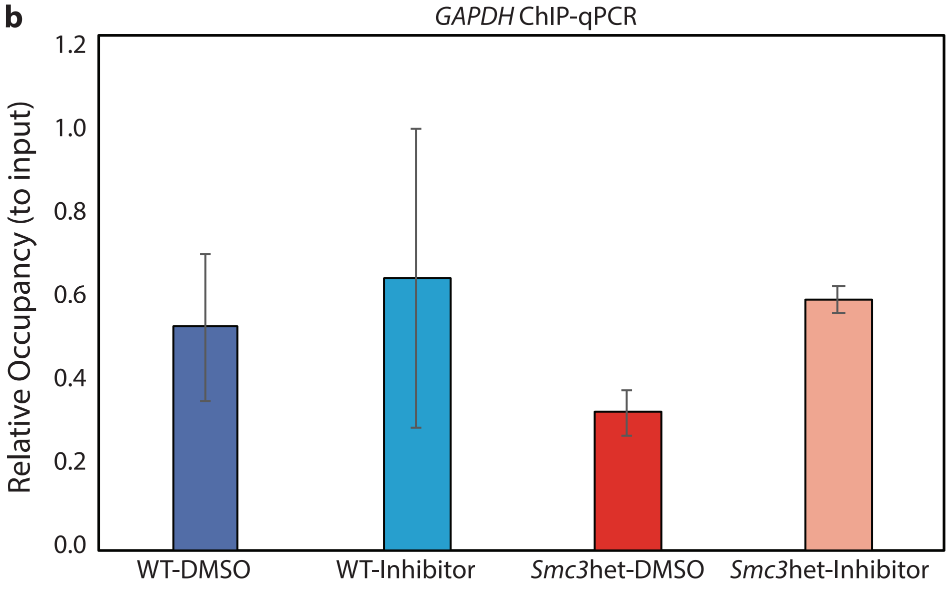

Supplemental Figure 3. a) Western blot showing H3K79me2 and total H3 levels amongst cells infected with pLKO or Rad21 shRNAs and treated with the DOT1L inhibitors or the vehicle. Quantification shown beneath. Error bars represent the standard error of the mean, and statistical significance between individual data points was determined using Student's T-test (two tailed, unpaired). # = $p < 0.1$, * = $p < 0.05$ **b)** H3K79me2 occupancy at GAPDH locus. No differences are significant via the Student's T-test (two tailed, unpaired), n = 3.

Supplemental Figure 4

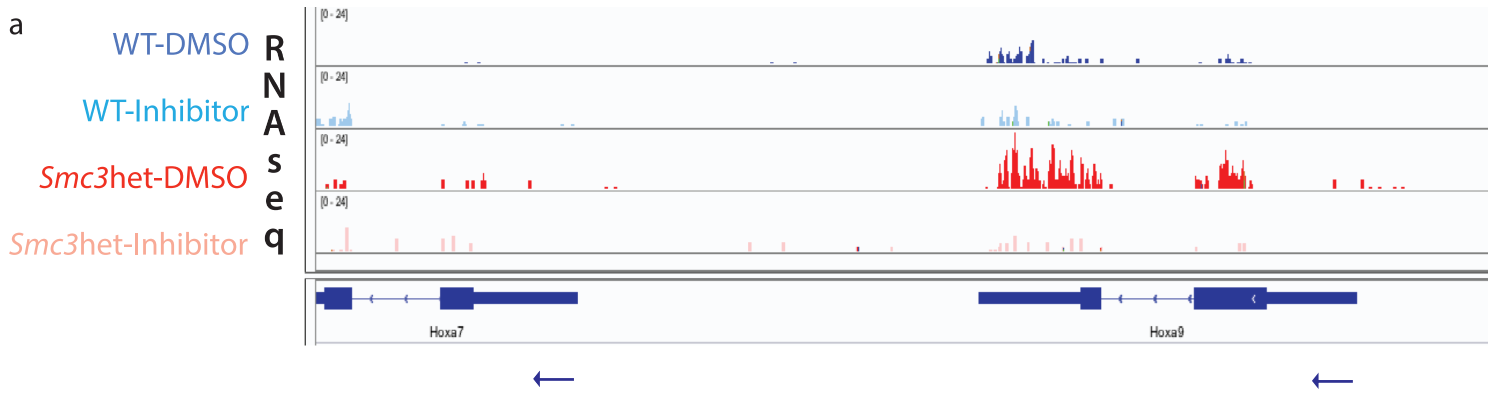

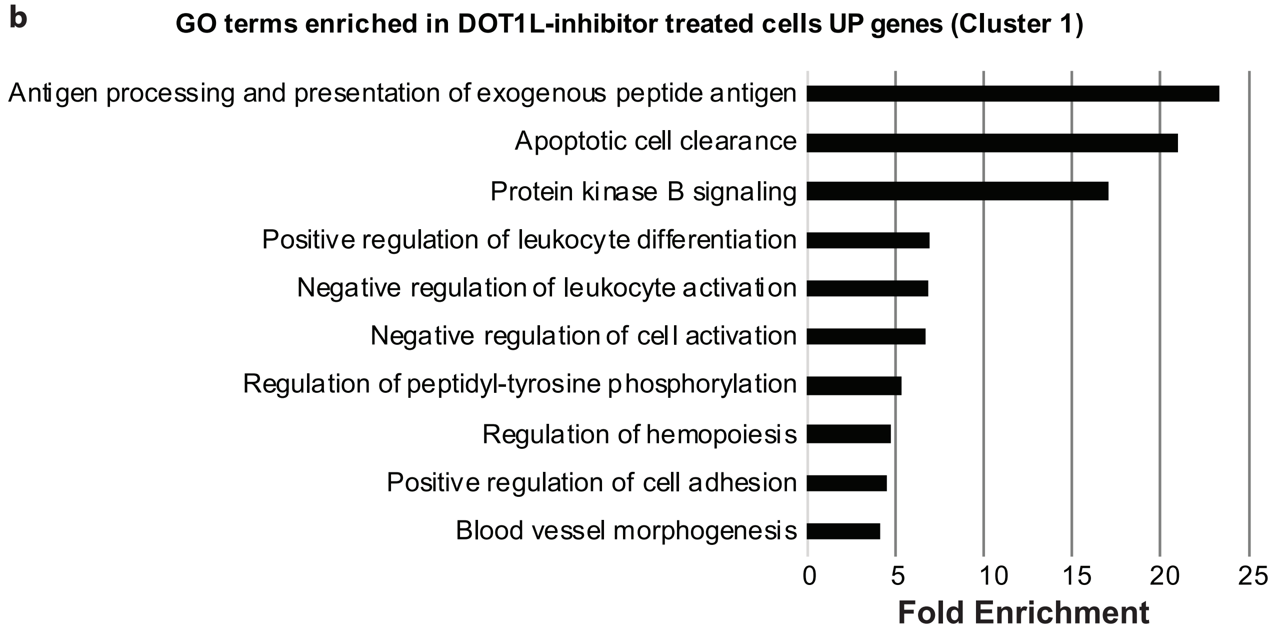

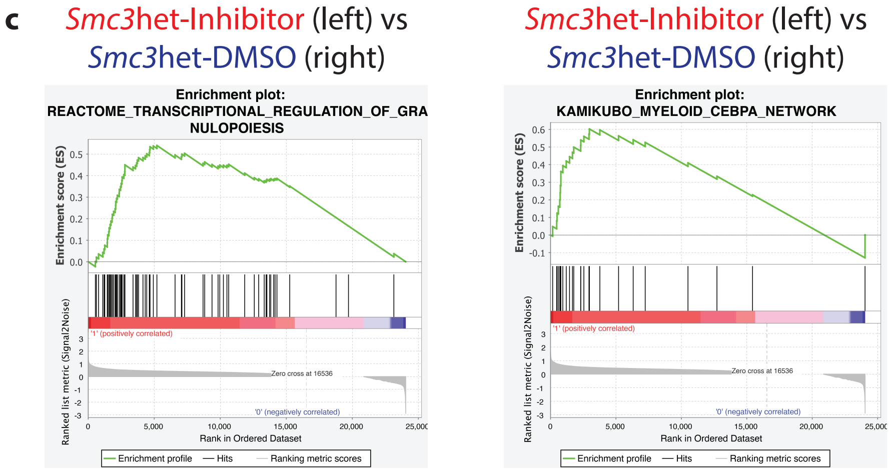

NES: +1.93
p-val: <0.001
FDR: 0.016

NES: +1.83
p-val: <0.001
FDR: 0.048

Supplemental Figure 5. a) IGV figure of the HoxA7/A9 genomic region and the expression as measured by RNAseq for either WT or Smc3het cells treated with the vehicle (DMSO) or DOT1L inhibitor (EPZ-5676). The X-axis indicates genomic position and the Y-axis indicates normalized feature counts. **b)** The top ten GO terms enriched within Cluster 1 (Figure 4) were identified based upon their fold enrichment. Normalized enrichment score (NES), p-value, and False discovery rate (FDR) are shown below. **c)** Gene Set Enrichment Analysis (GSEA) for highly enriched genesets when comparing RNAseq data for Smc3het cells treated with vehicle (DMSO) or DOT1L inhibitor (EPZ5676). Geneset names are listed on each enrichment plot, and statistics derived from GSEA are below.

Supplemental Figure 5

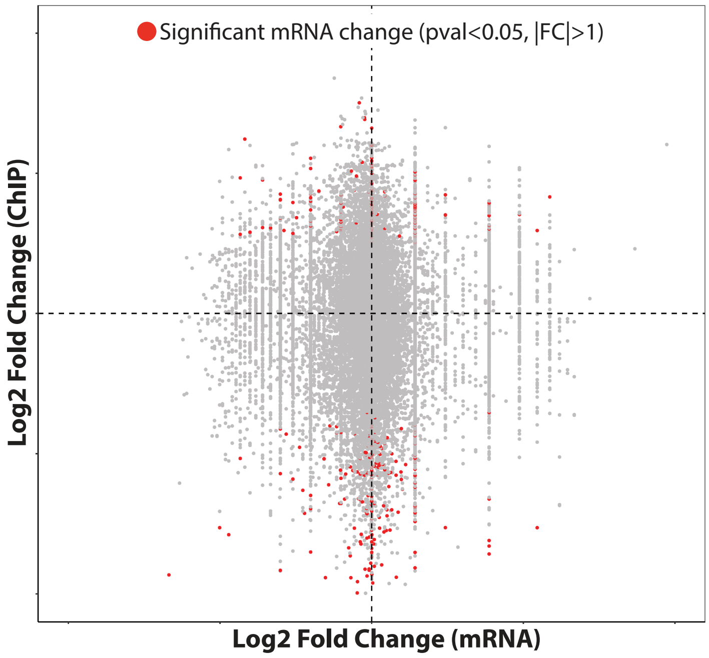

Supplemental Figure 5. The Log2 fold change for feature counts (RNAseq, X-axis) and H3K27me3 tag counts (ChIPseq, Y-axis) for all well-annotated (RefSeq, mm10) genes. Dots in red indicate significantly changed mRNAs as determined by p-val <0.05; |Log2FC|>1.

Supplemental Figure 6

a Total H3 Blot used in Figure 3b (1sec)

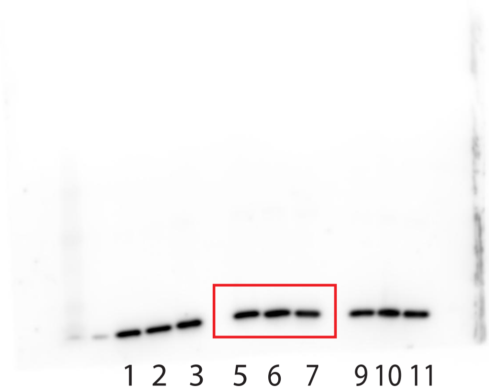

b H3K79me2 Blot used in Figure 3b (1sec)

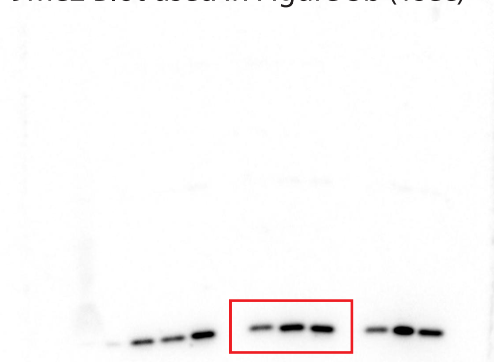

Lanes for a -d:
1: Empty Vector 1
2: *Rad21* shRNA #1 1
3: *Rad21* shRNA #2 1
4: empty lane
5: Empty Vector 2
6: *Rad21* shRNA #1 2
7: *Rad21* shRNA #2 2
8: empty lane
9: Empty Vector 3
10: *Rad21* shRNA #1 3
11: *Rad21* shRNA #2 3

c Total H3 Blot used in Figure 3b (10sec)

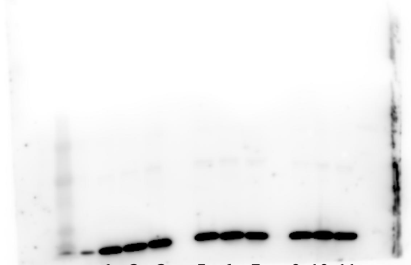

e H3K79me2 Blot used in Figure 3b (10sec)

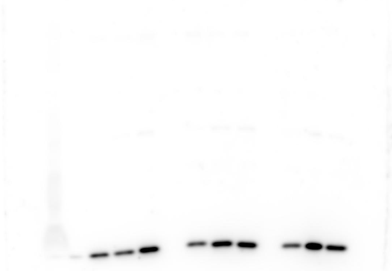

Lanes for e + f:
1: Empty Vector 1 - DMSO
2: Empty Vector 1 - 4777
3: Empty Vector 1 - 5676
4: Empty Vector 2 - DMSO
5: Empty Vector 2 - 4777
6: Empty Vector 2 - 5676
7: empty lane
8: *Rad21* shRNA #1 1 - DMSO
9: *Rad21* shRNA #1 1- 4777
10: *Rad21* shRNA #1 1- 5676
11: *Rad21* shRNA #1 2- DMSO
12: *Rad21* shRNA #1 2- 4777
13: *Rad21* shRNA #1 2- 5676
14: *Rad21* shRNA #1 3 - DMSO
15: *Rad21* shRNA #1 3 - 4777
16: *Rad21* shRNA #1 3 - 5676
17: empty lane
18: *Rad21* shRNA #2 1 - DMSO
19: *Rad21* shRNA #2 1 - 4777
20: *Rad21* shRNA #2 1 - 5676
21: *Rad21* shRNA #2 2 - DMSO
22: *Rad21* shRNA #2 2 - 4777
23: *Rad21* shRNA #2 2 - 5676

d Total H3 Blot used in Supplemental Figure3a

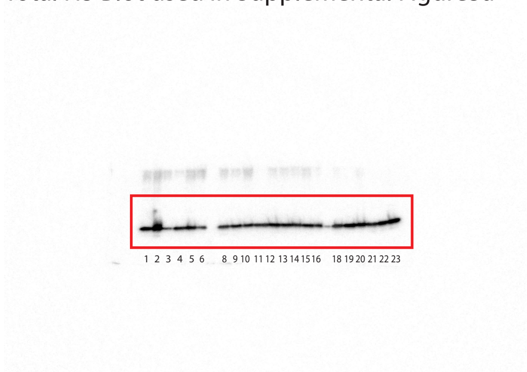

f H3K79me2 Blot used in Supplemental Figure 3a

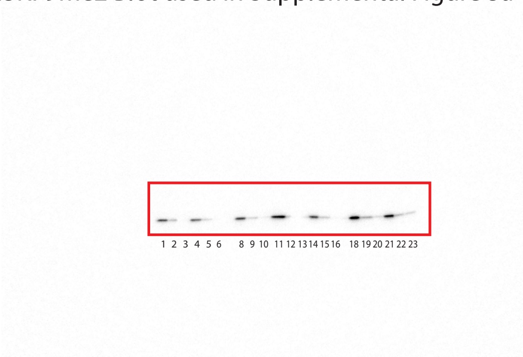

Supplemental Figure 6. Whole western blots for: Total H3 (a, c) and H3K79me2 (b,d) in *Rad21* Depleted cells versus Empty Vector cells at 1 sec (a, b) and 10sec (c, d) exposure. Total H3 (c) and H3K79me2 (d) in Dot1L Inhibitor treated (4777 or 5676) *Rad21*-depleted and Empty Vector cells. Red boxes indicate the parts of the blots used in Figure 3b (only including one replicate from a and b of this figure) and Supplemental Figure 3a (all lanes included from d and e of this figure).

Supplemental Table 1. Primers sequences used in this study.

Primer Target	Sequence	Purpose
HoxA9	GTCCCACGCTTGACACTCAC	RT-qPCR Forward
HoxA9	GAGCGAGCATGTAGCCAGT	RT-qPCR Reverse
HoxA7	CGCCTCCTACGACCAAAACA	RT-qPCR Forward
HoxA7	CTTCCTGTCGGGTCCTGAAC	RT-qPCR Reverse
Dot1L	CTTAACAACTACGAGCCCTTCT	RT-qPCR Forward
Dot1L	CAGGTCATCCTCTGTCATCTTG	RT-qPCR Reverse
β-actin	GATCTGGCACCACACCTTCTACAATG	RT-qPCR Forward
β-actin	CGTACATGGCTGGGGTGTTGAAG	RT-qPCR Reverse
HoxA9	GGAATAGGAGGAAAAAACAGAAGAGG	ChIP-qPCR Forward
HoxA9	TGTATGAACCGCTCTGGTATCCTT	ChIP-qPCR Reverse
HoxA7	CTCTTCTGTTTCCCATCCTGGT	ChIP-qPCR Forward
HoxA7	GGCAATATCCGGGATCCACT	ChIP-qPCR Reverse
GAPDH	GGTCCAAAGAGAGGGAGGAG	ChIP-qPCR Forward
GAPDH	GCCCTGCTTATCCAGTCCTA	ChIP-qPCR Reverse

Supplemental Table 2. Antibodies used in this study.

Antibody target	Catalog number	Purpose
H2K27me3	Millipore-Sigma 07-449	ChIPseq
H3K79me2	abcam ab3594	Immunobloting + ChIP
Total H3	Active Motif 61277	Immunobloting
Donkey-Anti-Rabbit-HRP	Santa Crus SC-2313	Immunobloting

Supplemental Table 3. GSEA results for respective comparisons including the top 5. most enriched gene sets from the curated and oncogenic signature gene set databases.

Gene sets enriched in *Smc3het* -DMSO vs WT-DMSO

	Gene set	NES	nom p-value	FDR
oncogenic (c6.all.v6.2)	KRAS.50 UP.V1 UP	-1.59	0.006	0.032
	KRAS.AMP.LUNG UP.V1 DN	-1.42	0.000	0.091
	KRAS.300 UP.V1 UP	-1.40	0.013	0.070
	BRCA DN.V1 UP	-1.37	0.000	0.068
	KRAS.LUNG.BREAST UP.V1 DN	-1.15	0.123	0.329
c2.all.v6.2	REACTOME LIGAND GATED ION CHANNEL TRANSPORT	-2.12	0.000	0.020
	MIKKELSEN ES HCP WITH H3K27ME3	-1.79	0.000	0.386
	NIELSEN LIPOSARCOME UP	-1.76	0.019	0.329
	GUENTHER GROWTH SPERICAL VS ADHERENT UP	-1.62	0.013	0.729
	MA PITUITARY ETAL BS ADULT UP	-1.54	0.044	1.000

Gene sets enriched in WT-DMSO vs Smc3het-DMSO

Gene set	NES	nom p-value	FDR
MYC UP.V1 UP	1.58	0.000	0.128
SNF5 DN.V1 DN	1.54	0.000	0.213
CSR LATE UP.V1 UP	1.53	0.000	0.220
CORDENONSI YAP CONSERVED SIGNATURE	1.52	0.011	0.290
STK33 DN	1.50	0.001	0.384
KEGG RIBOSOME	1.85	0.000	0.162
REACOME PEPTIDE CHAIN ELONGATION	1.82	0.000	0.125
REACTOME 3 UTR MEDIATED TRANSLATIONAL REGULATION	1.82	0.000	0.095
SHIN B CELL LYMPHOMA CLUSTER 9	1.81	0.000	0.086
REACOME INFLUENZA VIRAL RNA TRANSCRIPTION AND REPLICATION	1.80	0.000	0.075

Gene sets enriched in *Smc3het*- Inhibitor vs *Smc3het* -DMSO

	Gene set	NES	nom p-value	FDR
oncogenic (c6.all.v6.2)	RB P107 DN.V1 DN	-1.63	0.000	0.104
	IL21 UP.V1 UP	-1.47	0.002	0.648
	MTOR UP.N4.V1 UP	1.46	0.001	0.689
	ATM DN.V1 DN	-1.45	0.005	0.761
	SIRNA EIF4GI UP	-1.45	0.017	0.766
c2.all.v6.2	WANG IMMORTALIZED BY HOXA9 AND MEIS1 UP	-1.94	0.000	0.072
	REACTOME SRP DEPENDENT COTRANSLATIONAL PROTEIN TARGETING TO MEMBRA	-1.90	0.000	0.072
	REACTOME INFLUENZA VIRAL RNA TRANSCRIPTION AND REPLICATION	-1.90	0.000	0.049
	REACTOME METABOLISM OF RNA	-1.89	0.000	0.043
	REACTOME METABOLISM OF MRNA	-1.88	0.000	0.050

Gene sets enriched in *Smc3het* -DMSO vs *Smc3het* -Inhibitor

Gene set	NES	nom p-value	FDR
CAHOY NEURONAL	1.65	0.000	0.075
KRAS.3000 UP.V1 DN	1.65	0.000	0.038
KRAS.BREAST UP.V1 DN	1.62	0.000	0.035
KRAS KIDNEY UP.V1 UP	1.55	0.000	0.045
PRC2 EZH2 UP.V1 UP	1.42	0.000	0.149
MCMURRAY TP53 HRAS COOPERATION RESPONSE DN	2.13	0.000	0.035
PICCALUGA ANGIOIMMUNOBLASTIC LYMPHOMA UP	1.94	0.000	0.210
NIELSEN MALIGNANT FIBROUS HISTIOCYTOMA DN	1.90	0.003	0.206
SMID BREAST CANCER RELAPSE IN LUNG DN	1.84	0.000	0.297
REACTOME NCAM1 INTERACTIONS	1.82	0.003	0.277

Gene sets enriched in *Smc3het* -Inhibitor vs WT-Inhibitor

	Gene set	NES	nom p-value	FDR
oncogenic (c6.all.v6.2)	AKT UP.V1 UP	-1.71	0.000	0.040
	LEF1 UP.V1 UP	-1.61	0.000	0.062
	PIGF UP.V1 DN	-1.57	0.000	0.062
	KRAS.LUNG UP.V1 DN	-1.47	0.003	0.179
	P53 DN.V1 DN	-1.45	0.000	0.175
c2.all.v6.2	VALK AML CLUSTER 7	-2.08	0.000	0.010
	WELCH GATA1 TARGETS	-2.08	0.000	0.005
	MIKKELSEN NPX HCP WITH H3K4ME3 AND H3K27ME3	-1.99	0.000	0.017
	MEISSNER NPC HCP WITH H3K4MW3 AND H3K27ME3	-1.96	0.000	0.023
	KIM ALL DISORDERS DURATION CORR DN	-1.93	0.000	0.035

Gene sets enriched in WT-Inhibitor vs *Smc3het* -Inhibitor

Gene set	NES	nom p-value	FDR
ERB2 UP.V1 DN	1.83	0.000	0.001
PIGF UP.V1 UP	1.76	0.000	0.003
JAK2 DN.V1 DN	1.66	0.000	0.009
TBK1.DF DN	1.61	0.000	0.016
MTOR UP.N4.V1 DN	1.53	0.000	0.042
ZHANG TLX TARGETS 36HR DN	2.44	0.000	0.000
MILI PSEUDOPODIA HAPTOTAXIS UP	2.25	0.000	0.000
ZHANG TLX TARGETS SN	2.12	0.000	0.000
PYEON CANCER HEAD AND NECK VS CERVICAL UP	2.10	0.000	0.000
DACOSTA UV RESPONSE VIA ERCC COMMON DN	2.06	0.000	0.001

Gene sets enriched in *Cohesin-mutant vs Cohesin-WT*

	Gene set	NES	nom p-value	FDR
oncogenic (c6.all.v6.2)	VEGF_A_UP.V1_DN	1.61	0.000	0.031
	ERB2_UP.V1_DN	1.51	0.000	0.015
	CSR EARLY UP.V1 UP	1.57	0.000	0.019
	PIGFUP.V1 UP	1.53	0.000	0.032
	TBK1.DF DN	1.47	0.000	0.067
c2.all.v6.2	REACTOME TRANSLATION	2.02	0.000	0.000
	REACTOME 3 UTR MEDIATED TRANSCLATIONAL REGULATION	1.99	0.000	0.000
	BILANGES SERUM AND RAPAMYCIN SENSITIVE GENES	1.97	0.000	0.000
	KEGG RIBOSOME	1.96	0.000	0.000
	REACTOME GLUCURONIDATION	1.94	0.000	0.000

Gene sets enriched in Cohesin-WT vs Cohesin-mutant

Gene set	NES	nom p-value	FDR
RPS14 DN.V1 UP	1.94	0.000	0.000
RAF UP.V1 UP	1.57	0.000	0.124
KRAS.50 UP.V1 UP	1.56	0.000	0.087
SINGH KRAS DEPENDENCY SIGNATURE	1.53	0.039	0.079
MEK UP.V1 UP	1.44	0.000	0.123
MCLACHLAN DENTAL CARIES U[	2.24	0.000	0.000
KEGG INTESTINAL IMMUNE NETWORK FOR IGA PRODUCTION	2.14	0.000	0.000
VALK AML CLUSTER 5	2.11	0.000	0.002
REACTOME BETA DEFENSINS	2.04	0.000	0.009
REACTOME DEFENSINS	2.02	0.000	0.012

Supplementary Information: Materials and Methods

Primary Bone Marrow Isolation and Viral Transduction

Mice were euthanized in accordance with Medical College of Wisconsin Institutional Animal Care and Use Committee guidelines (IACUC# AUA00002688). To collect bone marrow, the tibiae and femurs of 6-8 week old C57BL/6 mice were crushed, filtered, and collected in EDTA buffer (PBS, 1% Pen/Strep, 0.5% Fetal Bovine Serum, 0.5mM EDTA). Red blood cells were removed using Red Cell Lysis Buffer (Sigma-Aldrich Cat #R7757). HSPCs were isolated using a lineage depletion kit according to the manufacturer's recommended protocol (Miltenyi, Cat# 130-090-858). HSPCs were cultured in StemPro medium (Gibco, Cat # 10640-019), IL-3 (10ng/mL, Miltenyi 130-099-510), IL-6 (10ng/mL, Miltenyi 130-096-684), and SCF (50ng/mL, Miltenyi 130-101-698) for 16 hours on suspension culture dishes. HSPCs were collected and transduced with lentivirus containing *Rad21* shRNA or Empty Vector (pLKO.1) control, similar to as previously described (**Figure** 1A, details in (1). Retronectin (Clontech, Cat# T100B) coated plates were preloaded with bone marrow medium (IDMD, 15% Fetal Bovine Serum, 1% Pen/Strep) containing *Rad21*-shRNA or empty vector constructs by centrifugation at 2000RPM for 60 minutes. Additional lentivirus was added to the HSPCs and they were transferred to the preloaded plates and centrifuged at 2000RPM for 90 minutes at 35°C. The plates were incubated at 37°C/5% CO_2 for 16 hours. Puromycin at 1 mcg/ml final

concentration was added to select for transduced cells for 24 hours prior to FSC/SSC sorting to isolate live cells.

Methylcellulose Colony Forming Assays

Live, virally transduced HSPCs were plated in methocult medium (StemCell Technologies, Cat # 173434) containing puromycin (1 mcg/ml), and 10μM DOT1L inhibitor (EPZ-4777 or EPZ-5676) or vehicle (DMSO) and incubated at 37°C/5% CO2 for 7 days (as previously described (1)). Representative images were taken of the colonies using an EVOS fluorescent microscope (Model # EVOS-F1) and camera. Two independent researchers determined colony numbers and the average is reported. To start the next passage, the cells were collected in bone marrow wash buffer (HBSS, 1% Pen/Strep, 2% FBS), counted using a hemocytometer, and 1,000 cells were plated into methocult containing puromycin (1 mcg/ml) and 10μM DOT1L inhibitor (EPZ-4777 or EPZ-5676) or vehicle (DMSO). This process was repeated for all subsequent passages.

Reverse Transcriptase and Quantitative Realtime PCR

RNA was collected from cells using Trizol reagent (Thermofisher, Cat# 15596-026), then converted to cDNA using an iScript cDNA synthesis kit (Biorad Cat# 170-8890). Realtime qPCR reactions were set up using Sybr Green Master mix using the indicated primer sets (**Supplemental Table 1).** Reactions were then run on a QuantStudio 6

realtime PCR machine (Applied Biosystems 4485699) and quantified by calculating the delta delta CT (n = 3 technical replicates).

Immunoblotting:

Cells were collected, washed with PBS, and resuspended in ice-cold RIPA buffer containing protease inhibitors. Protein concentration was determined using a BCA kit (Thermofisher Scientific, Cat# 23225), and equal amounts of proteins were loaded onto acrylamide gels. Separated proteins were transferred to PVDF membranes, blocked with 5% nonfat milk in Tris-buffered saline and Tween-20 (TBST) buffer for 1 hour at room temperature and incubated with primary antibodies (**Supplemental Table 2**) overnight. The blots were washed in TBST 3 times quickly followed by three 5 minutes washes in TBST at room temperature followed by incubation with secondary antibodies (**Supplemental Table 2**) for 1 hour at room temperature. The blots were again washed 3 times quickly followed by three 5 minutes washes in TBST at room temperature. After incubation with chemiluminescent substrate (GE Healthcare Cat # RPN2232) the blots were exposed to photofilm (MidSci Cat #BX810), which was developed in a Konica developer (Model #SRX-101A). Densitometry was performed using ImageJ analysis software.

Statistical Analyses

Number of replicates and statistical methods used in each figure can be found in the corresponding figure legends. Indicators of significant p-values are as follows: # = $p < 0.1$, * = $p < 0.05$, ** = $p < 0.01$, *** = $p < 0.001$.

Smc3 Mouse Model Bone Marrow Isolation

For sequencing studies, a mouse model of *Smc3* haploinsufficiency (2) was used instead of lentiviral knockdown of *Rad21. Smc3*$^{+/+}$ and *Smc3*$^{+/floxed}$ mice were treated with polyI:polyC (pIpC; 7 doses, every other day) to induce excision beginning at 4 weeks of age. 4 weeks post completion of pIpC treatment, mice were euthanized and HSPCs were isolated as stated above. HSPCs were then plated at a density of 1,000 live cells (determined by Trypan blue staining) per plate in methocult with puromycin (1 mcg/ml) and 10μM DOT1L inhibitor (Epizyme 5676 only) or vehicle (DMSO).

Chromatin Immunoprecipitation (ChIP) and ChIP-sequencing:

After 7 days culture in methocult, cells were fixed by resuspension in PBS containing 1% methanol-free formaldehyde, followed by incubation at room temperature for 10 minutes. Fixation was halted by addition of glycine followed by subsequent washes with PBS containing protease inhibitors (Sigma-Aldrich, Cat# P8340) and phenolmethylsulfonyl fluoride (PMSF). The fixed cells were resuspended in lysis buffer (0.1% SDS, 10mM EDTA, 150 M Tris-HCl, pH 8.1, .2mM PMSF, 1 ug/ml aprotinin,

1ug/mL leupeptin) and chromatin was sheared to 100-600bp fragments using a Diagenode Bioruptor Pico (Part # 141104). Input chromatin was saved (10% from each of the three WT-DMSO conditions) for comparison and sheared chromatin was incubated with primary antibodies overnight (**Supplemental Table 2**). Bound chromatin was collected by incubation with Dynabeads Protein A magnetic beads (Thermofisher Scientific, Cat #10001D) for 2 hours at 4C on a rotator. The bound chromatin was washed once each with low salt buffer (.1% SDS, 1% Triton-X100, 2nM EDTA, 20mM Tris-HCl pH 8.1, 150 mM NaCl), high salt buffer (.1% SDS, 1% Triton-X100, 2nM EDTA, 20mM Tris-HCl pH 8.1, 500 mM NaCl), LiCl wash buffer (.25M LiCl, 1% NP40 substitute, 1% deoxycholic acid, 1mM EDTA, 10 mM Tris-HCl pH 8.1), and twice with TE (10mM Tris-HCl pH 8.0, 1mM EDTA). The bound chromatin was eluted from the beads and the crosslinks were reversed by an overnight incubation at 65°C in SDS elution buffer (1% SDS, 10mM EDTA, 50mM Tris-HCl pH 8.0) shaking at 550rpm. The DNA was purified using phenol/chloroform extraction and isopropyl precipitation. ChIP-qPCR was performed by DNA input into each reaction. ChIP-qPCR primers can also be found in **Supplemental Table 1**, reactions were run in triplicate on the QuantStudio as described above. Instead of calculating ddCT, dCTs were calculated, comparing all samples to the input. ChIPseq libraries were made using the NEBNext Ultra II DNA Library Prep Kit (NEB #E7645S) per manufacturer protocol, using 3-120ng DNA as starting material. Library integrity was verified using the Agilent 4200

TapeStation for size distribution (~400-500bp) and qPCR (NEBNext Library Quant Kit #E7630) for concentration. Sequencing was performed on an Illumina NextSeq (single-end, 75 cycles). Preliminary analysis was performed using BasepairTech (basepairtech.com), an online workflow-based NGS analysis. Reads were aligned to mm10 and trimmed using Bowtie2 then peaks were called using MACS2, all according to default parameters. Aligned files were subjected to Deeptools3.1.1 bamCoverage analysis to visualize data in IGV. BasepairTech was used to call peaks using MACS2 and Deeptools3.1.1 (computeMatrix, plotHeatmap, plotProfile) was used to examine the genomic H3K27me3 landscape. Additionally, the DiffBind package was used to compare the peaksets identified by MACS2 and assess them for differential binding and generate the flower Venn diagram. Data can be found in GEO (Accession # GSE140361).

RNA-sequencing

RNA was collected from cells after 7 days in methocult using Trizol (Thermofisher, Cat# 15596-026) and libraries were made with the NEBNext Ultra RNA Library Prep Kit (#E7350) utilizing an ERCC spike-in (ThermoFisher 4456740)to increase rigor for downstream expression quantification. Library integrity was verified using the Agilent 4200 TapeStation for size distribution (~300-400bp) and qPCR (NEBNext Library Quant Kit #E7630) for concentration. Sequencing was performed on an Illumina NextSeq (paired-end, 38 cycles each). Bioinformatics was perfomed using BasepairTech. Reads

were aligned using STAR, expression was counted using Cufflinks, and comparisons between samples were made using DESeq, all with default parameters. Downstream clustering analysis was performed in R, following a Bioconductor workflow, RNA-seq workflow: gene-level exploratory analysis and differential expression (https://www.bioconductor.org/packages/devel/workflows/vignettes/rnaseqGene/inst/doc/rnaseqGene.html). Gene Set Enrichment Analysis (GSEA) was also performed. Data can be found in GEO (Accession # GSE140361).

TARGET patient data

Data from NCI's Therapeutically Applicable Research to Generate Effective Treatments (TARGET) study for AML was downloaded for 10 distinct patients with cohesin mutations (14 total datasets, 4 patients had both primary and recurrent disease data) and 49 patients without cohesin mutations. We performed DESeq2 to identify differentially expressed genes and then ran GSEAs on the differentially expressed gene profiles.

References:

1. Fisher JB, Peterson J, Reimer M, Stelloh C, Pulakanti K, Gerbec ZJ, et al. The cohesin subunit Rad21 is a negative regulator of hematopoietic self-renewal through epigenetic repression of Hoxa7 and Hoxa9. Leukemia. 2017;31(3):712-9.
2. Viny AD, Ott CJ, Spitzer B, Rivas M, Meydan C, Papalexi E, et al. Dose-dependent role of the cohesin complex in normal and malignant hematopoiesis. J Exp Med. 2015;212(11):1819-32.
